# Supplementary figures and images for: A novel mouse model of Campylobacter jejuni enteropathy and diarrhea
Source: PLoS Pathog. 2018 May 23;14(3):e1007083. doi: 10.1371/journal.ppat.1007083 (PMC5988333; doi:10.1371/journal.ppat.1007083)

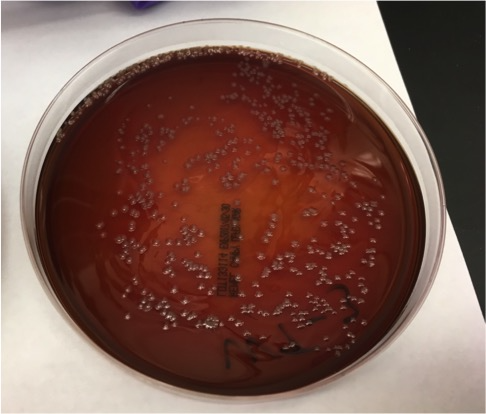

Supplement: S1 Fig — Image shown is representative of findings from 8 infected mice. (TIF) [file ppat.1007083.s001.tif]

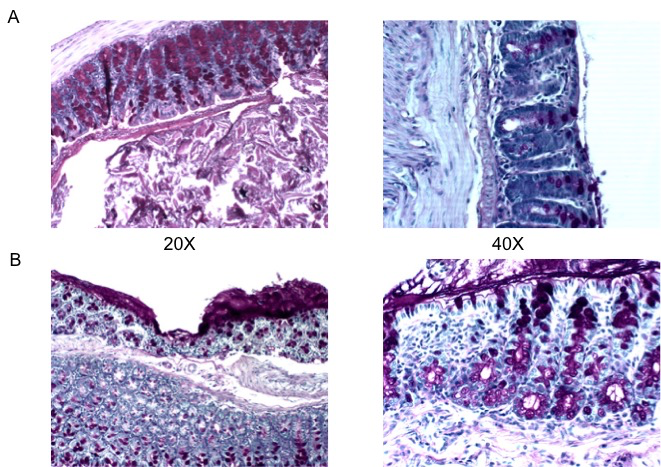

Supplement: S2 Fig — Notable changes in the zinc deficient mice were increased lamina propria cellular infiltrate and striking lumenal mucus discharge with only minor mucus discharge seen in house chow fed mice. (TIF) [file ppat.1007083.s002.tif]

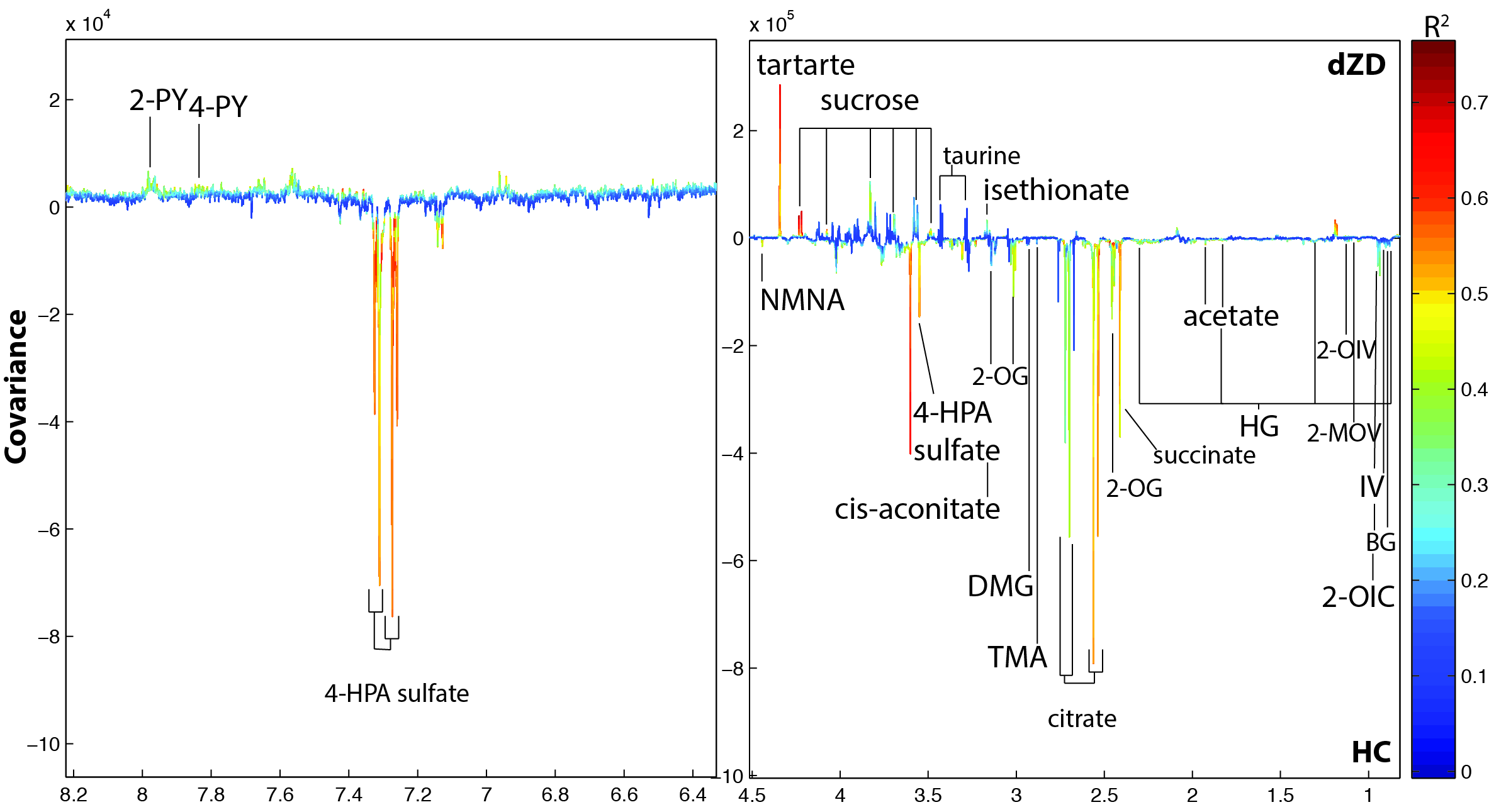

Supplement: S3 Fig — (HC: n = 8, dZD n = 8, (Q2Y = 0.45, P = 0.005) on day 0. Positive peaks correspond to metabolites being excreted in higher amounts by the dZD-fed mice and negative peaks indicate metabolites excreted in lower amounts as, compared to the HC-fed mice. Abbreviations: 2-OG, 2-oxoglutarate; 2-OIV, 2-oxoisovalerate; 2-OIC, 2-oxoisocaproate; 2-MOV, 3-methyl-2-oxovalerate; 2-PY, N-methyl-2-pyridone-5-carboxamide; 4-HPA, 4-hydroxyphenylacetate; BG, butyrylglycine; DMG, dimethylglycine; HG, hexanoylglycine; IV, isovalerate; NMNA, N-methyl-nicotinic acid; NMND, N-methylnicotinamide; TMA, trimethylamine. (TIF) [file ppat.1007083.s003.tif]
